# An antibody free approach to probe the presence of poly-ubiquitin chains on *C. elegans* sperm derived organelles after fertilization

**DOI:** 10.17912/micropub.biology.000972

**Published:** 2023-09-19

**Authors:** Justine Cailloce, Fanny Husson, Vincent Galy, Jorge Merlet

**Affiliations:** 1 Sorbonne Université, CNRS, Institut de Biologie Paris Seine, IBPS, Developmental Biology Laboratory, UMR7622, Paris, France

## Abstract

Upon *C. elegans* ’s oocyte fertilization, the sperm brings mitochondria and membranous organelles (MOs) which are rapidly eliminated by autophagy. Their poly-ubiquitylation is suspected to be a signal for their recognition and degradation but mitochondria poly-ubiquitylation remains debated. Using fluorescent Tandem-repeated Ubiquitin-Binding Entities (TUBEs) we confirmed the presence of K48- and K63-ubiquitin chains on MOs contrasting with the absence of signal on sperm mitochondria. This new and sensitive approach confirmed the poly-ubiquitylation of the MOs while providing additional arguments for the absence of substantial poly-ubiquitylation of sperm-derived mitochondria, suggesting that K63- and K48-poly-ubiquitylation are unlikely acting as a common targeting signal for their degradation.

**Figure 1. Poly-ubiquitin chains labelling on sperm derived organelles in early C. elegans embryos:**
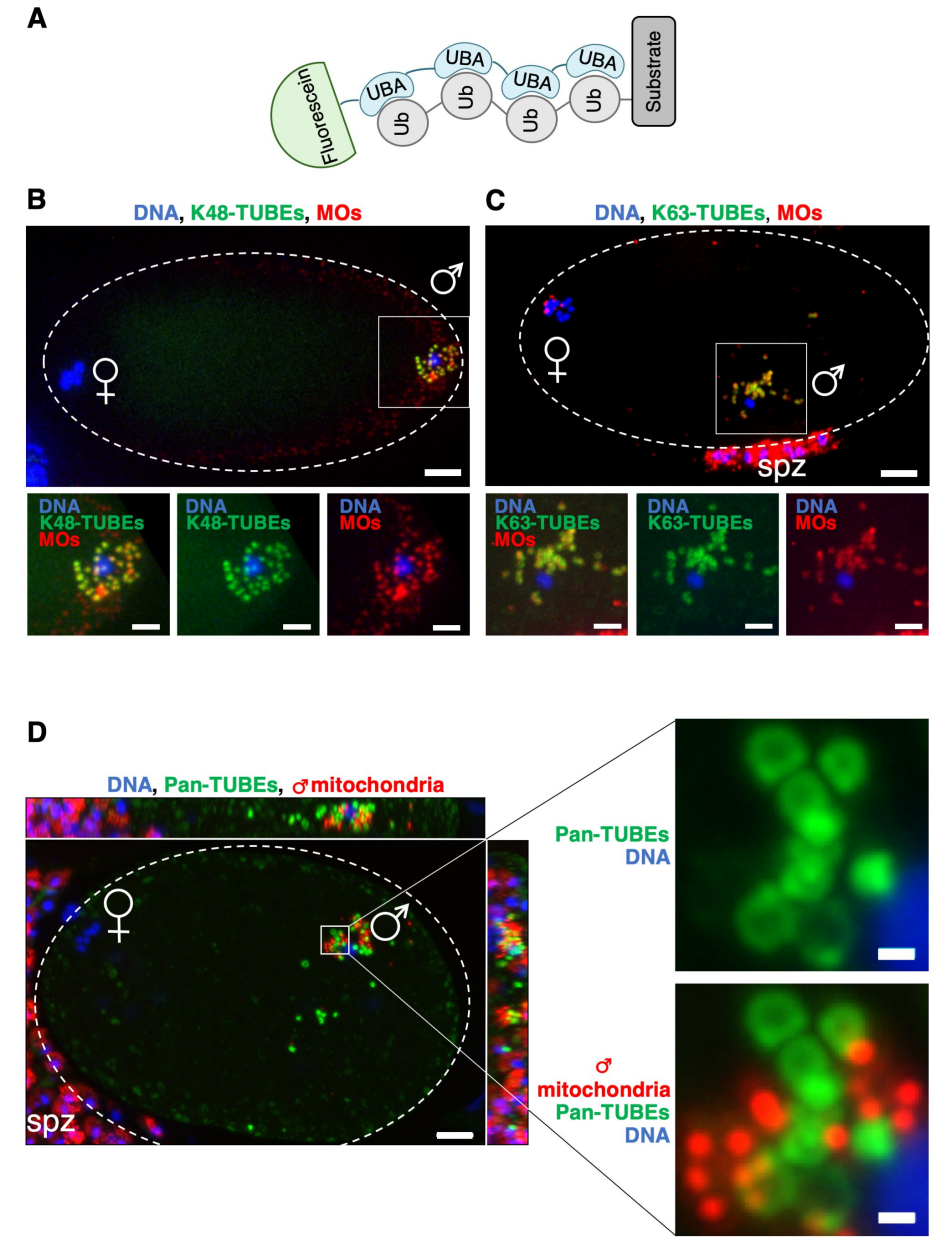
**(A)** Scheme of the Tandem-repeated Ubiquitin-Binding Entities (TUBEs) fused to fluorescein used to localize and visualize poly-ubiquitin chains. TUBEs are tetramerized ubiquitin association (UBA) domains with flexible poly-glycine links which bind poly-ubiquitin chains on substrates. Early *C. elegans* embryos labelled K48-TUBEs ( **B** ) or K63-TUBEs ( **C** ) (Green) showed a strong poly-ubiquitylation on MOs (red) for both K48- and K63-poly-ubiquitin chains. **(D)** In contrast, no poly-ubiquitin signal with Pan-TUBEs probes (green) was observed on sperm derived mitochondria (red) in early *C. elegans* embryos. The green rings observed are most likely poly-ubiquitylated MOs. Scale bars represent 5 µm in full embryo images and 2 µm in inserts. ♀︎and ♂ signs on images mark the presence of the respective pronuclei. Spz indicates spermatozoa outside the embryo. All images are maximum intensity projection images.

## Description

In *C. elegans,* like in most animal species, mitochondria and their genomes are inherited from the mother via oocyte’s mitochondria transmission. This uniparental transmission occurs despite the entry of sperm mitochondria and their genomes upon fertilization of the oocyte. Several mechanisms participate to specifically remove the spermatozoid contribution. Among the most conserved ones are the reduction of the sperm mtDNA load by a mitochondrial endonuclease and the specific recognition and degradation of sperm mitochondria in the oocyte via proteolytic pathways involving autophagy factors (Al Rawi et al. 2011; Sato and Sato 2011; DeLuca and O’Farrell 2012; Politi et al. 2014; Herpin et al. 2015). *C. elegans* relies on both types of mechanisms with a major contribution of allophagy, a specific type of autophagy. Interestingly in *C. elegans* , allophagy targets simultaneously the sperm mitochondria and other nematode specific sperm organelles : the Membranous Organelles (MOs) (Al Rawi et al. 2011; Sato and Sato 2011) . MOs are Golgi-derived membranous structures that partially fuse with the plasma membrane during the maturation of spermatocytes into mature spermatozoa (Wolf et al. 1978; Ward et al. 1981) . Both sperm organelles are rapidly eliminated after fertilization. However, the recognition signal(s) remain(s) unknown.

The implication of the poly-ubiquitylation of mitochondrial proteins in the recognition of the sperm-derived mitochondria was proposed in several mammalian species (Sutovsky et al. 1999) . Furthermore, since poly-ubiquitylation of mitochondria is a known mark for mitophagy in somatic cells (Kwon and Ciechanover 2017) the possible role of ubiquitin have been investigated. Several independent studies tested this hypothesis in *C. elegans* but no definitive conclusions were reached. Using specific anti-poly-ubiquitin antibodies, MOs but not the sperm mitochondria were described as poly-ubiquitylated (Al Rawi et al. 2011; Sato and Sato 2011; Molina et al. 2019) after their entry in the oocyte. While a newer study, using over-expressed GFP::ubiquitin fusion protein, described a very faint GFP::ubiquitin signal on sperm mitochondria compared to the bright GFP::ubiquitin signal surrounding the MOs (Sato et al. 2018) .

In order to clarify if sperm mitochondria are decorated with poly-ubiquitin chains that could serve as a signal for the recruitment of the autophagy machinery, we used a highly sensitive and specific antibody free approach (Hjerpe et al. 2009) to analyze the poly-ubiquitylation status of sperm organelles in the 1 cell stage *C. elegans* embryos. Tandem-repeated Ubiquitin-Binding Entities (TUBEs) consist of tetramerized Ubiquitin-Binding Associated (UBA) domains separated by flexible linkers ( Figure 1A ). They show a high affinity for poly-ubiquitin chains (Hjerpe et al. 2009) . Different types of TUBEs have been generated that recognize all poly-ubiquitin chains (Pan-TUBEs) but also specifically K48 or K63 chains (Hjerpe et al. 2009; Sims et al. 2012) . About 20 different families of domains able to bind ubiquitin have been described which allows to discriminate within the ubiquitin chain structure diversity and complexity observed *in vivo* . Thus, the K48-TUBEs have 100-fold preference for K48 linkage and K63-TUBEs >1 000-fold preference for K63 linkage over other linkage types. We used TUBEs fused to fluorescein as an alternative to antibodies and GFP::ubiquitin strains to probe for the presence of poly-ubiquitylated proteins on sperm organelles after fertilization.

We set up a protocol to label early *C. elegans* embryos with TUBEs. MOs visualized using a specific antibody, were clearly labeled with the K48- and K63-poly-ubiquitin specific probes ( Figure 1B and 1C). These results confirmed previous observations (Al Rawi et al. 2011; Sato and Sato 2011; Hajjar et al. 2014; Molina et al. 2019) validating the use of TUBEs to detect poly-ubiquitylated proteins in *C. elegans* . Interestingly, since no additional signal was seen beside the MO-associated signal, this was strongly suggesting the absence of K48 and K63 poly-ubiquitin chains associated with sperm mitochondria. This was confirmed using the Pan-TUBEs probe and a more resolutive and sensitive microscope. As shown in Figure 1D, while the Pan -ubiquitin fluorescent probe nicely decorated structures corresponding to the MOs, we did not detect any signal colocalizing with the fluorescently labeled sperm mitochondria. In addition, the Pan-TUBEs did not label any structure on mature spermatozoa outside the embryo ( Figure 1D ), confirming also previous observations showing that poly-ubiquitylation on MOs only occurs after their entry in the oocyte (Al Rawi et al. 2011) . These experiments demonstrate that in the one cell stage embryo, while MOs are massively poly-ubiquitylated, the sperm mitochondria are not poly-ubiquitylated or at a sub-detectable level.

Probing the presence of poly-ubiquitin chains in early *C. elegans* embryos using TUBEs represent a new sensitive and specific method to label endogenous poly-ubiquitin chains. It allowed us to confirm that before the recruitment of the autophagy machinery, MOs and sperm derived mitochondria are not equivalent regarding poly-ubiquitylation. MOs are highly poly-ubiquitylated whereas sperm mitochondria do not present any detectable signal. Therefore, our results suggest that the poly-ubiquitylation of a mitochondrial component is unlikely or at a very low level. TUBEs may therefore become instrumental in future studies to interfere with the degradation of poly-ubiquitylated proteins and test the impact on the clearance of MOs and sperm-derived mitochondria and the functional consequences *in vivo* . It must be noted that even if the Pan-TUBEs should recognize all types of poly-ubiquitin chains and not only K48 and K63 chains, as well as mono-ubiquitylation or multiple mono-ubiquitylation when they are the main population (Hjerpe et al. 2009) , we cannot exclude that, in the cellular context of the early *C. elegans* embryo, the recognition of other poly-ubiquitin chains K6, K11, K27, K29, K33 or M1 would only be accomplished by a specific construction and not by the Pan-TUBEs we used. Therefore, even if we cannot completely rule-out that poly-ubiquitylation could be involved in targeting sperm-derived mitochondria to the allophagy machinery the molecular nature of the signal(s) harbored by sperm mitochondria will have to be identified using genetic or biochemical approaches.

## Methods

*C. elegans* strains and maintenance

Two different *Caenorhabditis elegans* strains were used, the wild-type Bristol N2 strain and the CU607 strain ( *smIs23 [ pkd-2 ::gfp] II; him-5 ( e1490 ) V* ). CU607 contains the *him-5 ( e1490 )* allele which allows a higher frequency of males in the population and the *smIs23* transgene which allows a male specific GFP expression in neurons (not required in this study). Both strains were maintained under standard conditions at 15° or 20°C on Nematode Growth Media plates seeded with OP50 bacteria (Brenner 1974) .

CMXRos staining of male mitochondria

For red fluorescence staining of mitochondria, young adult males CU607 were cultivated for 12 to 15 hours on NGM plates containing Mitotracker Red CMXRos (1 µg/mL), seeded with OP50 bacteria and protected from light.

Staining and Immunofluorescence

Males with CMXRos labelled mitochondria were mated overnight at 15°C with young N2 adult hermaphrodites. Gravid hermaphrodites were then dissected on poly-L-lysine coated glass slides (poly-L-lysine 1 g/L, gelatin 2%, chromium III 0.2 g/L, sodium azide 1 mM) in meiosis buffer (Leibowitz L-15 60%, inulin 0.5 mg/mL, HEPES 25 mM (pH=7.4), FBS 20%). Embryos were permeabilized by freeze crack, fixed in cold methanol for 20 minutes at -20°C, washed with PBS-T (PBS with 0.1% Tween 20) and blocked in PBS-T-milk (5% milk). Embryos were washed in PBS-T and incubated in a wet chamber protected from light for 2 hours at room temperature in PBS-T containing fluorescein labelled TUBEs (1/1000). For MOs staining, SP56 anti-MO (1/100) was added together with TUBEs, slides were washed in PBS-T and incubated for 1 h at room temperature with anti-mouse Alexa 633 (1/800). Slides were then washed twice in PBS-T, once in PBS and mounted in Vectashield® antifade mounting medium with DAPI (Vector) to label DNA. Finally, slides were covered with a coverslip and sealed with Va-La-P (Vaselin-Lanolin-Parafin, 1:1:1) (Askjaer et al. 2014) .

Images Acquisitions

Embryos with TUBEs and MOs staining were imaged on a Zeiss Cell Observer spinning disk microscope equipped with a Yokogawa CSU-X1 spinning disk head, an Evolve EM CCD camera and a 1.46 NA 100X oil-immersion objective. Embryos with TUBEs and CMXRos labelled mitochondria were imaged on a Zeiss Axio Observer Z1 equipped with an Apotome module, an Axiocam 506 monochrome cooled CCD camera and a 1.4 NA 100X oil-immersion objective. Maximum intensity Z-projections from multichannel stacks of images every 0.4 µm (spinning disk) or 0.24 µm (Apotome) are presented. All images were processed with Zen software (blue edition).

## Reagents

List of strains

**Table:** 

STRAIN	GENOTYPE	AVAILABLE FROM
N2		CGC
CU607	*smIs23* [ pkd-2 ::gfp] II *; him-5 ( e1490 )* V	Gift from Dr. D. Xue (Univ. Colorado, USA) (Peden et al. 2007)

List of Antibodies

**Table:** 

ANTIBODY	ANIMAL AND CLONALITY	DESCRIPTION
Anti-MO (SP56)	Mouse mono-clonal antibody	Recognize MOs, gift from S. Strome (Univ. of California Santa Cruz, USA) (Ward et al. 1986)
Goat anti-mouse Alexa 633	Goat polyclonal antibody	FisherScientific # A-21050

List of reagents/chemicals

**Table:** 

NAME	SOURCE	IDENTIFIER
PAN selective TUBEs-(TUBEs 2 fluorescein)	LifeSensors	UM-0502F-0050
K63-TUBEs-fluorescein	LifeSensors	UM-0504F-0050
K48-TUBEs-fluorescein	LifeSensors	UM-0507F-0050
MitoTracker Red CMXRos	FisherScientific	# 11569106
